# Parkinsonism following Bee Sting: A Case Report

**DOI:** 10.1155/2012/476523

**Published:** 2012-10-10

**Authors:** Ruchika Mittal, Sanjay Munjal, Dheeraj Khurana, Ashok Gupta

**Affiliations:** ^1^Speech and Hearing Unit, Department of Otolaryngology and Head and Neck Surgery, Post Graduate Institute of Medical Education and Research, Chandigarh 160 012, India; ^2^Department of Neurology, Post Graduate Institute of Medical Sciences and Education and Research, Chandigarh 160 012, India; ^3^Department of Otolaryngology and Head and Neck Surgery, Post Graduate Institute of Medical Education and Research, Chandigarh 160 012, India

## Abstract

We are reporting here a rare case of Parkinsonism (Hypokinetic dysarthria) caused after a bee stung, a member of the hymenoptera order. The main aim of this report is to orient the clinicians with the possibility of extrapyramidal syndromes because of hymenoptera stings.

## 1. Introduction 

Parkinsonism is a syndrome that encompasses the symptoms of resting tremor, rigidity, akinesia (paucity of movement), and postural instability. Communication disorders are common in Parkinsonism, often beginning with a decrease in vocal loudness and at times progressing to more severe functional limitations characterized by changes in speaking rate, articulatory precision, and speech intelligibility [1].

We are reporting here one of the relatively uncommon cases of Parkinsonism (Hypokinetic dysarthria) caused after being stung, a member of the hymenoptera order.

## 2. Case Report 

A 46-year-old female referred from the Department of Neurology to the Speech and Hearing Unit, Department of Otolaryngology and Head and Neck Surgery at PGIMER, Chandigarh, with chief complaint of difficulty in speaking. The detailed case history and the old records of the patient revealed that the patient developed an anaphylactic shock after being stung by a bee on her left fourth finger. She was admitted in the hospital within few hours. Next day onwards there was a decrease in all motor activities. A possibility of secondary Parkinsonism was considered and she responded to antiparkinsonism drugs (carbidopa + levodopa).

The speech and language assessment of the patient revealed hypokinetic dysarthria. The main features of her dysarthric speech were inappropriate pitch, breathy voice, laboured speech, prosodic insufficiency, and rate disturbances. The overall intelligibility of her speech was poor. The patient was scheduled for twice a week therapy and the aim was to improve the overall intelligibility of the speech. The techniques like relaxation exercises to improve the breathing pattern and movement, pacing technique to control the rate of speech, and adduction exercises to increase vocal intensity were used. Measures during and after therapy documented significant improvement in the overall intelligibility of her speech within two months. 

## 3. Discussion

The pathology of Parkinsonism is associated with the loss of dopaminergic neurons in the basal ganglia (especially the substantia nigra and brain stem). It is divided into subgroups depending upon its etiology and associated signs and symptoms. Idiopathic or primary Parkinson's disease (also known as paralysis agitans) is the term applied when the cause of the syndrome is unknown. Secondary Parkinsonism, which includes a number of disorders with extrapyramidal features that have an identifiable causal agent, some of which would include toxins, infections, drugs (neuroleptics), and reported trauma or multiple strokes. Another group of syndromes, known as Parkinsonism Plus, refers to heterogeneous system degeneration, such as progressive supranuclear palsy, striatonigral degeneration, Shy-Drager syndrome, or olivoponto cerebellar degeneration. Because these syndromes are associated with damage to multiple neural systems, the type of dysarthria associated with them may be different from that associated with Parkinsonism [1].

The literature was reviewed and interestingly enough there is only one case report of Parkinsonism after a wasp sting, a member of hymenoptera order to which bee also belongs too [2]. However, there are few case reports which document cerebral infarction and toxic reactions associated with hymenoptera stings [3–6].

## 4. Conclusion

Bee sting is very common and it has not been commonly associated with Parkinsonism. Therefore, we believe that the present work may draw the attention of the clinicians to include hymenoptera stings in the differential diagnosis of acute and chronic extrapyramidal syndromes.
